# The Whitish Inner Mantle of the Giant Clam, *Tridacna squamosa*, Expresses an Apical Plasma Membrane Ca^2+^-ATPase (PMCA) Which Displays Light-Dependent Gene and Protein Expressions

**DOI:** 10.3389/fphys.2017.00781

**Published:** 2017-10-10

**Authors:** Yuen K. Ip, Kum C. Hiong, Enan J. K. Goh, Mel V. Boo, Celine Y. L. Choo, Biyun Ching, Wai P. Wong, Shit F. Chew

**Affiliations:** ^1^Department of Biological Sciences, National University of Singapore, Singapore, Singapore; ^2^The Tropical Marine Science Institute, National University of Singapore, Singapore, Singapore; ^3^Natural Sciences and Science Education, National Institute of Education, Nanyang Technological University, Singapore, Singapore

**Keywords:** calcification, calcium, plasma membrane Ca^2+^-ATPase, symbiosis, tridacnid, zooxanthellae

## Abstract

Giant clams live in symbiosis with extracellular zooxanthellae and display high rates of growth and shell formation (calcification) in light. Light-enhanced calcification requires an increase in the supply of Ca^2+^ to, and simultaneously an augmented removal of H^+^ from, the extrapallial fluid where shell formation occurs. We have obtained the complete coding cDNA sequence of *Plasma Membrane Ca*^2+^*-ATPase* (*PMCA*) from the thin and whitish inner mantle, which is in touch with the extrapallial fluid, of the giant clam *Tridacna squamosa*. The deduced PMCA sequence consisted of an apical targeting element. Immunofluorescence microscopy confirmed that PMCA had an apical localization in the shell-facing epithelium of the inner mantle, whereby it can actively secrete Ca^2+^ in exchange for H^+^. More importantly, the apical PMCA-immunofluorescence of the shell-facing epithelium of the inner mantle increased significantly after 12 h of exposure to light. The transcript and protein levels of *PMCA*/PMCA also increased significantly in the inner mantle after 6 or 12 h of light exposure. These results offer insights into a light-dependable mechanism of shell formation in *T. squamosa* and a novel explanation of light-enhanced calcification in general. As the inner mantle normally lacks light sensitive pigments, our results support a previous proposition that symbiotic zooxanthellae, particularly those in the colorful and extensible outer mantle, may act as light-sensing elements for the host clam.

## Introduction

**Figure 1:**
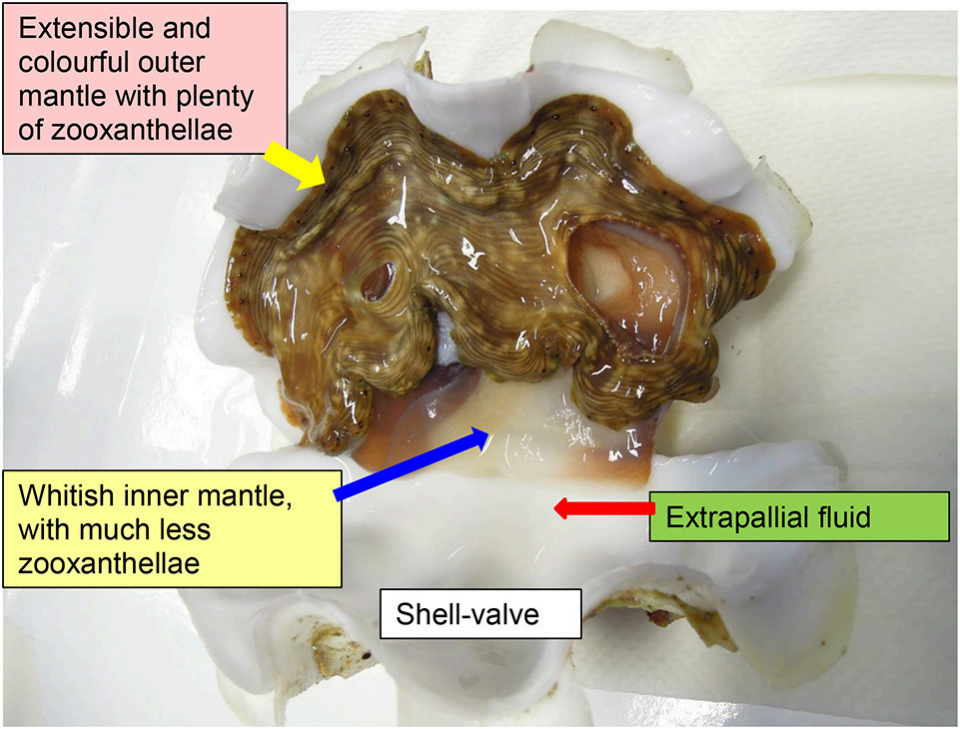
An illustration on the outer mantle and inner mantle of the giant clam *Tridacna squamosa*. The extensible, fleshy, and colorful outer mantle harbors extracellular zooxanthellae in tertiary tubules, while the thin and whitish inner mantle is in direct contact with the extrapallial fluid and participates in shell formation.

There is a general theme of metabolic interaction in alga–invertebrate symbioses. In general, the heterotrophic animal host supplies inorganic nutrients to the photoautotrophic symbiotic zooxanthellae, which in turn donate photosynthate to the host to support its energy and nutrient demands. Giant clams are alga-invertebrate associations, belonging to Phylum: Mollusca, Class: Bivalvia, Order: Veneroida, Family: Cardiidae, and Subfamily Tridacninae. They live in symbiosis with zooxanthellae (*Symbiodinium*; particularly, Clade A, C, and D), and can be found along coral reefs in the tropical Indo-Pacific. The symbiotic zooxanthellae reside extracellularly in a branched tubular system inside the host clam. Microscopic tertiary tubules are located mainly in the surface tissue of the fleshy and colorful outer mantle, which is extensible and retractable (Figure 1). The symbiotic zooxanthellae residing in these tertiary tubules are well-positioned to receive sufficient light for photosynthesis. Besides pigments, the outer mantle has iridophores which consist of small groups of cells (iridocytes) containing stacks of tiny platelets (Griffiths et al., 1992). The reflective platelets scatter light of photosynthetically productive wavelengths into the tissue while back-reflecting non-productive wavelengths (Holt et al., 2014). Thus, the extensible outer mantle is brightly colored. By contrast, the inner mantle adjacent to the extrapallial fluid, as demarcated by the pallial line of the shell-valve, is thin and whitish (Figure 1), and is involved in shell formation (calcification). With contributions from the symbiotic zooxanthellae, giant clams can grow at high rates in nutrient deficient tropical seawater, although the rates of shell formation (Klumpp and Griffith, 1994; Watanabe and Oba, 1999) and growth (Lucas et al., 1989) are critically dependent on the availability of light. In fact, the Sr/Ca ratio in the shell of the giant clam, *Tridacna derasa*, exhibits striking diurnal variations attributable to the daily cycle of light-enhanced calcification (Sano et al., 2012).

Calcification in bivalve molluscs involves the deposition of calcium carbonate, based on the reaction Ca^2+^ + ${\text{HCO}}_{3}^{-}$⇔ CaCO_3_ + H^+^, from the extrapallial fluid onto the inside surface of each shell-valve. Consequently, the removal of H^+^ would promote supersaturation conditions resulting in a more rapid precipitation of CaCO_3_ as aragonite. As expected, Ip et al. (2006) reported a significant increase in the pH of the extrapallial fluid in the fluted giant clam, *Tridacna squamosa*, exposed to light. Their results indicated that H^+^ could combine with NH_3_ to form ${\text{NH}}_{4}^{+}$ in the extrapallial fluid, and ${\text{NH}}_{4}^{+}$ could be transported subsequently into the shell-facing epithelial cells of the inner mantle to support glutamine production (Ip et al., 2006). It is possible that ${\text{NH}}_{4}^{+}$ transport involved a Na^+^/${\text{NH}}_{4}^{+}$-ATPase (Ip et al., 2015), but the molecular characterization and subcellular localization of such a transporter has not been confirmed.

The excess H^+^ produced during light-enhanced calcification must be removed in order to maintain whole-body acid-base balance. Recently, Hiong et al. (2017a) have obtained from the ctenidium (gill) of *T. squamosa* the complete cDNA sequence of a *Na*^+^*/H*^+^ *exchanger* 3 (*NHE3*)-*like transporter*, which regulates intracellular pH and ionic balance by mediating H^+^ efflux in exchange for Na^+^ uptake in a 1:1 stoichiometry. Immunofluorescence microscopy demonstrates that the NHE3-like transporter is localized to the apical membrane of epithelial cells of the ctenidial filaments and the tertiary water channels (Hiong et al., 2017a). Particularly, the immunofluorescence of the ctenidial filaments in clams exposed to 12 h of light is consistently stronger than that in the control kept in darkness. Light also induces significant increases in the transcript level and protein abundance of the NHE3-like transporter in the ctenidium of *T. squamosa*, indicating that its transcription and translation are light-dependent (Hiong et al., 2017a). This can be regarded as a response to augment H^+^ excretion in pursuance of whole-body acid-base balance during light exposure, denoting a collaboration between the ctenidium and the inner mantle in light-enhanced calcification. Furthermore, light stimulates giant clams to absorb and assimilate exogenous ammonia (Wilkerson and Trench, 1986; Fitt et al., 1993), and Hiong et al. (2017b) have cloned the complete coding cDNA sequence of a host *Glutamine Synthetase* (*GS*), which assimilates ${\text{NH}}_{4}^{+}$ into glutamine, from the ctenidium of *T. squamosa*. Like the NHE3-like transporter, the transcript level and protein abundance of *GS*/GS increase significantly after 12 h of light exposure, indicating that the absorbed ammonia is probably assimilated into glutamine.

Light-enhanced calcification necessitates an increase in CaCO_3_ precipitation besides the increased removal of H^+^. Therefore, there must be an augmentation of Ca^2+^ supply from the haemolymph to the extrapallial fluid where calcification occurs. However, the mechanisms involved in the light-enhanced transepithelial movement of Ca^2+^ through the thin layer of whitish inner mantle remain elusive. The intracellular Ca^2+^ concentration is highly regulated because Ca^2+^ is involved in the sensitive regulation of various cell signaling pathways. In general, the free Ca^2+^ concentration (~100 nmol l^−1^) in the cytosol of animal cells at resting is maintained much lower than that (~1.2 mmol l^−1^) in the extracellular fluid (Carafoli, 2002). The class of P-type ATPases involved in Ca^2+^ transport is designated P2 which comprises three families of transporters: plasma membrane Ca^2+^-ATPases (PMCAs), sarco(endo)plasmic reticulum Ca^2+^-ATPases (SERCAs), and secretory-pathway Ca^2+^-ATPases (SPCAs). Ca^2+^ activated ATPase activity attributable to a combination of these three types Ca^2+^-ATPases has been detected in the mantle of *T. squamosa* (Ip et al., 2015).

Specifically, PMCAs are high-affinity Ca^2+^ extrusion pumps present in virtually all eukaryotic cells. Its basic function is to expel intracellular Ca^2+^ in a highly regulated fashion so as to maintain the 10,000-fold Ca^2+^ concentration gradient across the plasma membrane (Blaustein et al., 2002). In mammals, four *PMCA* gene isoforms have been identified (Carafoli and Brini, 2000), and their translated proteins bear differences mainly in the N- and C-terminal regions (Guerini, 1998). Furthermore, the alternative splicing of transcripts produces many splice variants with differences mainly in their C-terminal amino acid residues and their affinities for Ca^2+^ and calmodulin (Strehler and Zacharias, 2001). In chick tibia, PMCA is present only in the basolateral membrane but absent from the ruffled border or clear zone of the osteoclast (Akisaka et al., 1988). In osteoblasts, PMCA can be localized to the basolateral membrane (Francis et al., 2002; Stains et al., 2002) or apical membrane (Nakano et al., 2004; Go and Korzh, 2013), and there are indications that PMCA takes part in intracellular Ca^2+^ homeostasis following signaling events (Stains et al., 2002).

Since no information is available on the role of PMCA in light-enhanced calcification in giant clams, this study was performed to clone the complete coding cDNA sequence of *PMCA* from the inner mantle of *T. squamosa*, and to examine whether the deduced PMCA amino acid sequence comprised an apical targeting element. Following the description of Hiong et al. (2017a), the portion of the thin and whitish mantle inside the pallial line is defined as the inner mantle, while the fleshy and brightly colored portion of the mantle outside the pallial line, which is unique to giant clams, is regarded as the outer mantle (Figure 1). The thin inner mantle comprises a shell-facing epithelium and a seawater-facing epithelium separated by loose connective tissues and haemolymph. It was hypothesized that PMCA would be localized to the apical membrane of the shell-facing epithelium of the inner mantle where it could pump Ca^2+^ into the extrapallial fluid and simultaneously remove H^+^ from it. Hence, immunofluorescence microscopy was performed using a custom-made anti-PMCA antibody to elucidate the subcellular localization of PMCA in the inner mantle. We also hypothesized that the expression levels of *PMCA*/PMCA in the shell-facing epithelium of the inner mantle, like those of the ctenidial NHE3-like transporter (Hiong et al., 2017a) and GS (Hiong et al., 2017b), could be light-dependent. Therefore, it was essential to determine quantitatively by ImageJ the integrated density of PMCA-immunofluorescence in the shell-facing epithelium, particularly along the apical membrane, of the inner mantle of *T. squamosa*. In addition, efforts were made to determine the transcript level and protein abundance of *PMCA*/PMCA in the inner mantle of *T. squamosa* in response to light exposure. It was expected that results obtained would provide new insights into the mechanisms of light-enhanced calcification in giant clams.

## Materials and methods

### Human and animal rights

No institutional (National University of Singapore Institutional Animal Care and Use Committee) approval was required for research on invertebrates including giant clams during the course of this study. To minimize pain, stress, and suffering, giant clams were anesthetized with 0.2% phenoxyethanol before killing for tissue sampling.

### Animals

Adult specimens of *T. squamosa* (521 ± 184 g; mean ± *SD*; *N* = 30) were procured from Xanh Tuoi Tropical Fish, Ltd (Vietnam) and kept in an indoor aquarium. They were maintained in three glass tanks with recirculating seawater. The water conditions and light intensity were the same as those described by Ip et al. (2015), except that temperature was kept at 26 ± 1°C.

### Experimental conditions and tissue collection

For molecular work, five giant clams were killed for tissue sampling at the end of a 12-h dark period, and they were regarded as controls (*N* = 5). Another 15 individuals were exposed to light and sampled after 3, 6, or 12 h of light exposure (*N* = 5 for each time point). In order to simulate the day/light hours experienced by the clams in their natural habitat, parallel controls were not adopted in this study so that no clam would be exposed to more than 12 h of darkness. The mantle of the anesthetized clam was dissected along the pallial line to obtain the whitish inner mantle. Excised tissue samples were blotted dry and immediately freeze-clamped with metal tongs pre-cooled by liquid nitrogen. The frozen samples were stored in –80°C until processing for molecular analyses. For immunofluorescence microscopy, tissue samples were collected from another 10 clams, of which 5 were exposed to darkness for 12 h and another 5 were exposed to light for 12 h (*N* = 5 each).

### Total RNA extraction and cDNA synthesis

The extraction and purification of total RNA from the inner mantle were performed using TRI Reagent^TM^ (Sigma-Aldrich, St. Louis, MO, U.S.A.) and RNeasy Plus Mini Kit (Qiagen, Hilden, Germany), respectively. A Shimadzu BioSpec-nano spectrophotometer (Shimadzu, Tokyo, Japan) was used to quantify the purified RNA. The integrity of the purified RNA was determined by electrophoresis. Four micrograms of purified RNA were used to synthesize first strand cDNA using a RevertAid^TM^ first strand cDNA synthesis kit (Thermo Fisher Scientific, Waltham, MA, U.S.A.).

### PCR, RACE PCR, and sequencing

The partial *PMCA* sequence was obtained from the inner mantle of *T. squamosa* using a set of primers (forward: 5′-GTVMGDCATYTDGATGCCTG-3′; reverse: 5′-CCRTAGGGTTTVCGTTBBAG-3′) designed according to the conserved regions of *Pinctada fucata PMCA* (EF121960.1), *Hyriopsis cumingii PMCA* (KR080192.1), *Stylophora pistillata PMCA* (AY360080.1), and *Strongylocentrotus purpuratus PMCA* (NM_001033650.1). PCR was performed using DreamTaq™ polymerase (Thermo Fisher Scientific Inc.) in a 9902 Veriti 96-well thermal cycler (Applied Biosystems, Carlsbad, CA, USA). The cycling conditions were similar to those of Hiong et al. (2017b). PCR products were separated by electrophoresis using 1% agarose gel. Products of the appropriate molecular mass were extracted from the gels using FavorPrep Gel Purification Mini Kit (Favorgen Biotech Corp., Ping-Tung, Taiwan), and cloned into pGEM®-T Easy vector (Promega Corporation). The ligated vector was transformed into JM109 competent cells and grown overnight on Luria-Bertani (LB) agar with ampicillin, X-gal and IPTG. The plasmids were extracted from selected white colonies using the resin-based plasmid miniprep kit (Axygen Biosciences, Union city, CA, USA). Multiple clones of PMCA fragments were sequenced bi-directionally. The fragments were verified to be *PMCA* through blasting with the Genbank database. Analyses of multiple clones confirmed the presence of only one form of *PMCA* in the inner mantle of *T. squamosa*. Based on the partial *PMCA* sequence, specific primers (forward: 5′-GGACAGAGTTGAGAAATTGGGCGTGG-3′; reverse: 5′-TGGGTATATCTGGTACCGACGTGGC-3′) were then designed to obtain the complete *PMCA* sequence of *T. squamosa* through multiple sequencing in both directions using 5′ and 3′ RACE (SMARTer™ RACE cDNA amplification kit: Clontech Laboratories, Mountain View, CA, USA).

Samples were prepared for sequencing using the BigDye® Terminator v3.1 Cycle Sequencing Kit (Thermo Fisher Scientific Inc.) followed by ethanol/sodium acetate precipitation. Sequencing was conducted with a 3130XL Genetic Analyzer (Thermo Fisher Scientific Inc.). Sequence assembly and analysis were performed using BioEdit version 7.2.5. The cDNA sequence of *PMCA* was deposited into GenBank (KU724109).

### Deduced amino acid sequence and phylogenetic analysis

The *PMCA* of *T. squamosa* was translated into the deduced amino acid sequence using the ExPASy Proteomic server (http://web.expasy.org/translate/). It was aligned with PMCA sequences selected from various animals using BioEdit to generate the sequence identity matrix in order to confirm its identity. The transmembrane regions (TM) of PMCA from *T. squamosa* were predicted using MEMSAT3 and MEMSAT-SVM provided by PSIPRED protein structure prediction server (http://bioinf.cs.ucl.ac.uk/psipred/). NetPhos 2.0 was used to identify the putative phosphorylation sites.

Various amino acid sequences of PMCA, SERCA, and SPCA were obtained from Genbank or UniProtKB/TrEMBL with the following accession numbers: *Crassostrea gigas* PMCA3 (EKC42567.1), *H. cumingii* PMCA (AKF17160.1), *P. fucata* PMCA (ABL63470.1), *Aplysia californica* SERCA (XP_012940855.1), *Biomphalaria glabrata* SERCA (XP_013087479.1), *C. gigas* SERCA (EKC33522.1), *Mizuhopecten yessoensis* SERCA (BAA37143.1), *P. fucata* SERCA (ABS19815.1), *Placopecten magellanicus* SERCA (AAC63909.1), *A. californica* SPCA1 (XP_012938577.1), *A. californica* SPCA2 (XP_005099465.1), *B. glabrata* SPCA1 (XP_013063771.1), *C. gigas* SPCA1 (EKC29072.1), and *Thelohanellus kitauei* SPCA1 (KII66755.1). These sequences were aligned with PMCA from *T. squamosa* using ClustalX2 and subjected to phenogramic analysis using the neighbor-joining method and 1,000 bootstrap replicates with Phylip in order to confirm the identity of PMCA of *T. squamosa*.

### Determination of mRNA expression by quantitative real-time PCR (qPCR)

RNA (4 μg) extracted from the tissue samples were reverse-transcribed using random hexamer primers with the RevertAid^TM^ first strand cDNA synthesis kit. The transcript level of *PMCA* was determined using specific forward (5′-TAGCCAAGTTTCTACAGTTCCA-3′) and reverse (5′-GTCCATTATCAAGTTCACCCA-3′) qPCR primers. The amplification efficiency for *PMCA* was 99.9%. Absolute quantification of transcripts in the qPCR reaction was performed following the methods described previously (Hiong et al., 2017a,b), and the quantity of transcripts was calculated using the plasmid standard curves. Triplicate analyses of each sample were performed using a StepOnePlus™ Real-Time PCR System (Thermo Fisher Scientific Inc.). Despite adopting the absolute quantification method in this study, efforts were made to verify that the transcript level of a reference gene (α-tubulin) was not light-dependent. Using a pair of specific qPCR primers (forward: 5′-GTGCCAAAGGATGTCAATGTC-3′; reverse: 5′-CTTAGCCATATCTCCGCCTG-3′), it had been confirmed that the mRNA expression level of α-tubulin remained unchanged throughout the 12 h of light exposure as compared to the control kept in darkness (results not shown).

### Antibodies

The anti-PMCA antibody was developed by GenScript (Piscataway, NJ, USA) and raised as a rabbit polyclonal antibody against residues 52-65 (SPNEGLPGHDADLE) of the PMCA amino acid sequence of *T. squamosa*. The epitope sequence was selected based on high antigenicity, hydrophilicity, and results from BLAST showing multiple hits of PMCA of different animals. The anti-α-tubulin antibody (12G10) used for Western blotting was obtained from the Developmental Studies Hybridoma Bank maintained by the University of Iowa, Department of Biological Sciences, Iowa City, IA 52242, USA. The commercially available anti-PMCA monoclonal antibody 5F10 was purchased from Thermo Fisher Scientific Inc.

### Immunofluorescence microscopy

Inner mantle samples were excised and immersion fixed overnight in 3% paraformaldehyde in seawater at 4°C, and processed according to the method of Hiong et al. (2017a). The paraffin-embedded samples were sectioned (3 μm) and collected on slides. Antigen retrieval was performed by treating deparaffinized sections with 0.05% citraconic anhydride and 1% sodium dodecyl sulfate solution. The section was blocked using the Pierce Fast Blocking Buffer (Thermo Fisher Scientific Inc.) for 10 min. Then, it was incubated with the custom-made anti-PMCA antibody raised against the PMCA of *T. squamosa* diluted in blocking buffer (1.67 μg ml^−1^) for 1 h at 37°C, followed with 1 h of incubation at 37°C with the secondary antibody, goat anti-rabbit Alexa Fluor 488 (2.5 μg ml^−1^; Life Technologies Corporation). A peptide competition test was performed to validate the specificity of the custom-made anti-PMCA antibody. The anti-PMCA antibody (25 μg) was incubated with the immunizing peptide (125 μg) provided by GenScript in a total volume of 200 μl for 1 h at 25°C. The resulting medium containing the antibody was diluted in Pierce Fast Blocking Buffer and used for immuno-staining. An Olympus BX60 epifluorescence microscope (Olympus Corporation, Tokyo, Japan) was used for viewing the sections, and images were captured using an Olympus DP73 digital camera (Olympus Corporation).

The quantification of total fluorescence intensity based on the custom-made anti-PMCA antibody raised against the PMCA of *T. squamosa* was performed on original images captured at 200 × magnification for the shell-facing epithelium of the inner mantle of giant clams kept in darkness (control) or exposed to light for 12 h, using Image J version 1.50i software with an Olympus Viewer Plugin (http://rsbweb.nih.gov.libproxy1.nus.edu.sg/ij/). Images were converted to grayscale. Six different regions, the summation of which represented at least 50% of the total area of the shell-facing epithelium of the inner mantle, were randomly chosen from each image for measurement. Regions of similar areas adjacent to the shell-facing epithelium with little fluorescence were selected for background subtraction. The area, integrated density, and mean gray value were used to calculate the total fluorescent intensity in both dark and light samples based on the method of Potapova et al. (2011). Results represent the integrated density of six random measurements for each individual image. A total of 10 individual images (*N* = 5 for control kept in darkness, and *N* = 5 for clams exposed to 12 h of light) were quantified.

Immunofluorescence microscopy was also performed using a commercially available mouse monoclonal anti-PMCA antibody 5F10 (1:400 dilution; Thermo Fisher Scientific Inc.) following the above-mentioned procedure, but with Pierce Superblock® blocking buffer (Thermo Fisher Scientific Inc.) instead of Pierce Fast Blocking Buffer. Alexa Fluor 568 goat anti-mouse secondary antibody (Life Technologies Corporation) was used at a dilution of 1:800 and the slides were viewed using Olympus WIG fluorescence filter (excitation, 520–550 nm; emission, >580 nm).

### Western blotting

Protein extraction and SDS-PAGE were performed employing the methods of Hiong et al. (2017a,b). No heating was applied to the sample as preliminary experiment revealed that P2 ATPases from *T. squamosa* were unstable at high temperature. One hundred micrograms of proteins were separated by SDS-PAGE (8% acrylamide for resolving gel, 4% acrylamide for stacking gel). Proteins were then electrophoretically transferred onto PVDF membranes using a transfer apparatus (Bio-Rad Laboratories). After transfer, Western blotting was performed using the Pierce Fast Western Blot kit, SuperSignal® West Pico Substrate (Thermo Fisher Scientific Inc.) according to the manufacturer's instructions with slight modifications. Briefly, the membranes were blocked with Pierce Fast Blocking Buffer for 15 min at 25°C. Then, the membranes were incubated with the custom-made anti-PMCA antibody raised against the PMCA of *T. squamosa* (0.67 μg ml^−1^) or anti-α-tubulin antibody (0.05 μg ml^−1^) in Fast Western Antibody Diluent for 1 h at 25°C and then with optimized anti-rabbit or anti-mouse horseradish peroxidase-conjugated secondary antibody for 15 min at 25°C. The membranes were washed six times with the Fast Western Wash Buffer provided in the kit in order to have a clear background. In order to validate the specificity of the anti-PMCA antibody raised against the PMCA of *T. squamosa*, a peptide competition test was also performed. After pre-incubation with the immunizing peptide as mentioned above, the neutralized anti-PMCA antibody was diluted in Fast Western Antibody Diluent and used for Western blotting as described above. Bands were visualized by chemiluminescence (provided by the Pierce Fast Western Blot kit) using X-ray films (CL-XPosure^TM^ Film; Thermo Fisher Scientific Inc.) as described by Hiong et al. (2017a). Results were presented as relative protein abundance of PMCA normalized with α-tubulin.

Western blotting was also performed using the anti-PMCA antibody 5F10 (Thermo Fisher Scientific Inc.) as described above, except that the membrane was blocked in Pierce Superblock® blocking buffer (Thermo Fisher Scientific Inc.) for 15 min at 25°C prior to incubation with 5F10 (1:500 dilution). Subsequently, bands were visualized by chemiluminescence using X-ray films.

### Statistical analysis

Results were presented as means ± standard errors of means (S.E.M.). The SPSS Statistics version 19 software (IBM Corporation, Armonk, NY, USA) was used for statistical analyses. Student's t-test was used to evaluate the difference between two means. Levene's test was applied to determine the homogeneity of variance for the data set. One-way analysis of variance (ANOVA) followed by either the Tukey or Dunnett T3 *post-hoc* test, depending on the homogeneity of variance of the data set, was used to evaluate differences among multiple means. Differences with *P* < 0.05 were regarded as statistically significant.

## Results

### *PMCA*/PMCA sequences and phenogramic analysis

**Figure 2:**
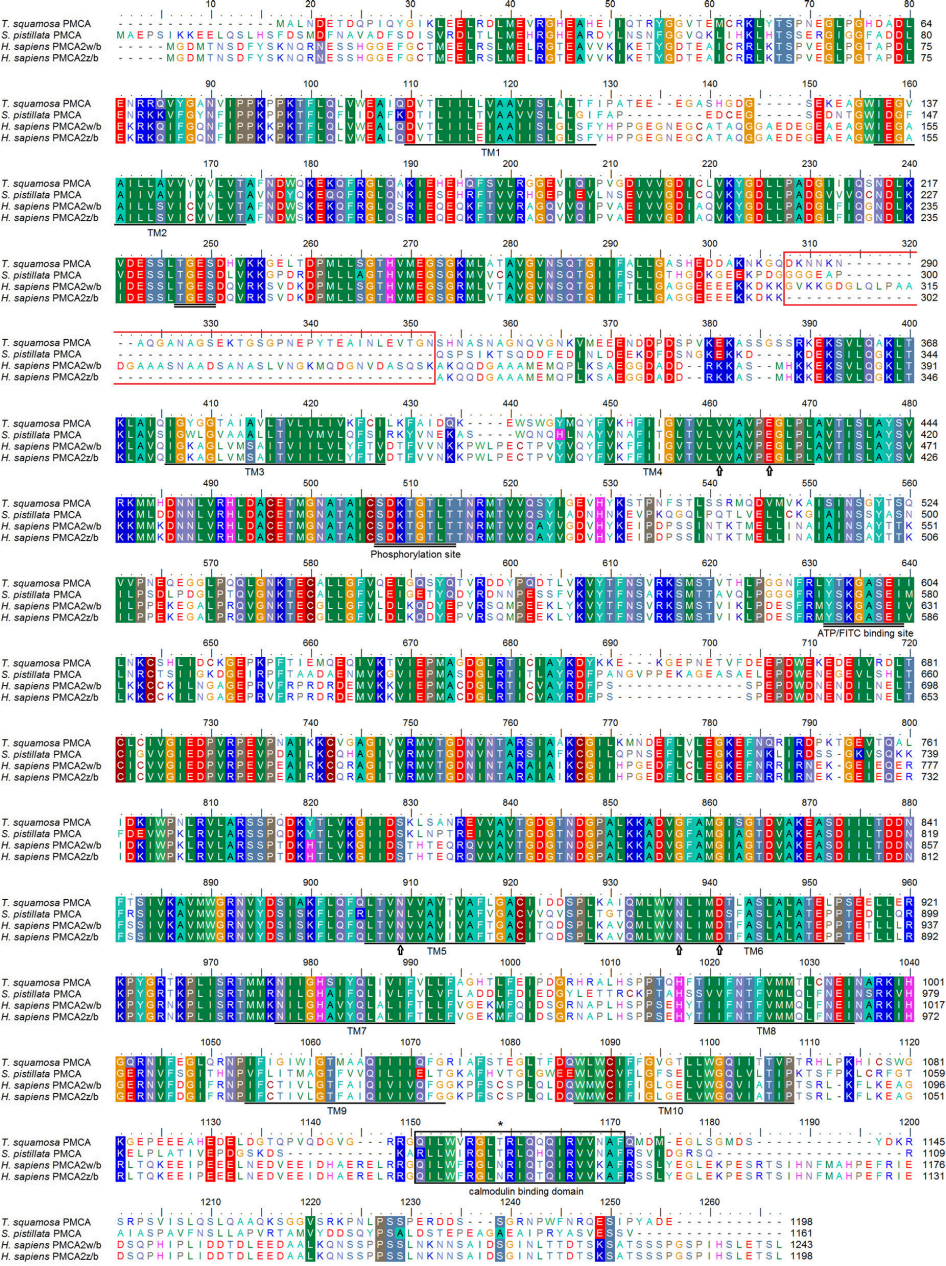
Molecular characterization of Plasma Membrane Ca^2+^-ATPase (PMCA) from the inner mantle of *Tridacna squamosa*. Multiple amino acid alignment of PMCA from the inner mantle of *T. squamosa*, with other known PMCA from *Stylophora pistillata* (AAR13013) and *Homo sapiens* (NP_001001331; NP_001674). Identical or similar amino acid residues are indicated by shaded residues. The conserved regions containing the TGES, DKTGTLT, and YTKGASEI sequence motifs are double underlined. The putative Ca^2+^-binding sites are marked with an open arrow. The region that gives rise to splice variants of PMCA2 in *H. sapiens* and the calmodulin-binding domain are indicated in red and black boxes, respectively. The asterisk denotes the amino acid residues phosphorylated by PKC. The 10 predicted transmembrane regions (TM1-TM10) are underlined. The transmembrane domains were predicted using MEMSAT3 and MEMSAT-SVM provided by PSIPRED protein structure prediction server.

**Figure 3:**
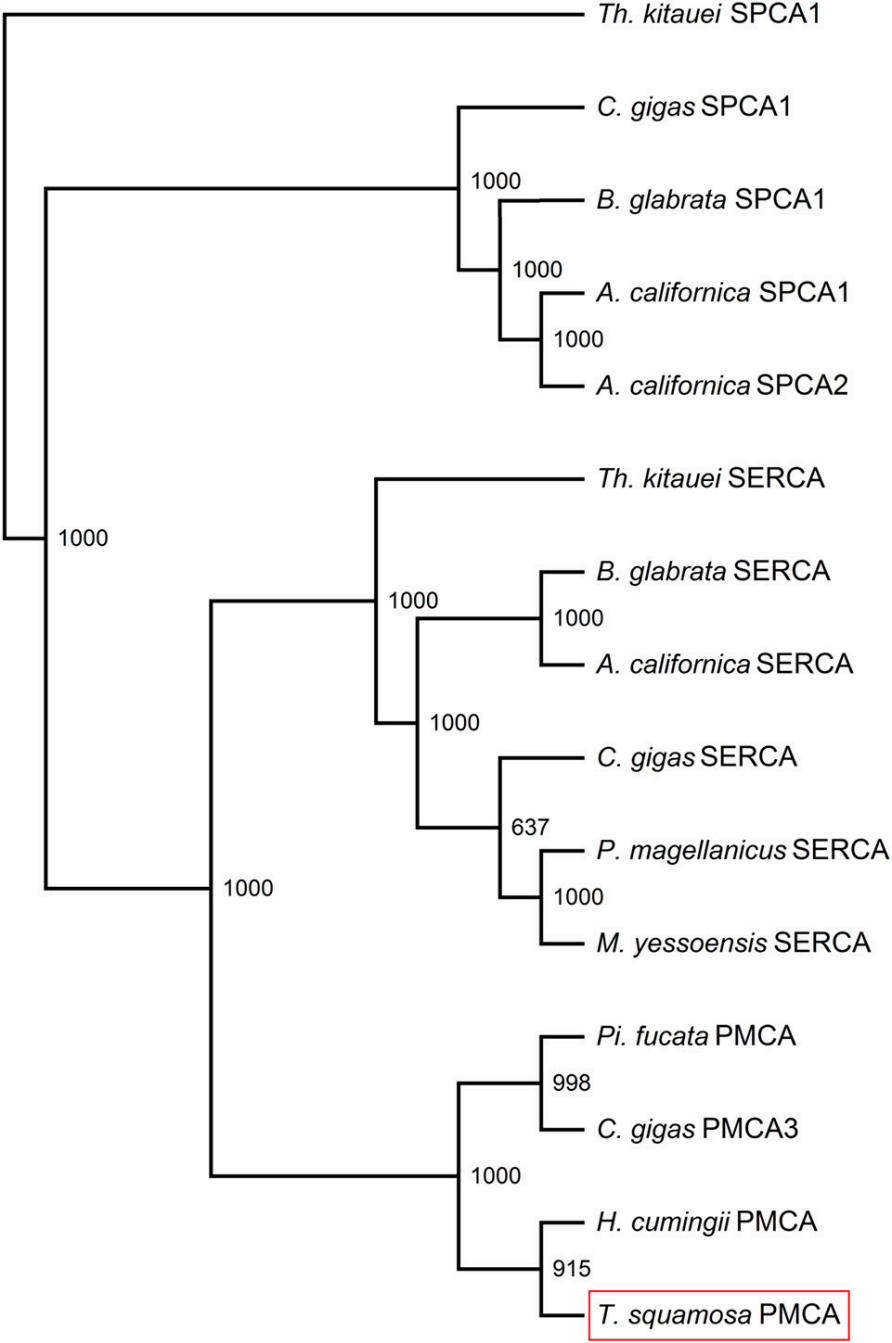
Phenogramic analysis of Plasma Membrane Ca^2+^-ATPase (PMCA) of *Tridacna squamosa*. Numbers presented at each branch point represent bootstrap values from 1,000 replicates. SPCA1 from *Thelohanellus kitauei* is used as the outgroup for the phenogramic analysis.

The *PMCA* cDNA coding sequence obtained from the inner mantle of *T. squamosa* comprised 3597 bp [GenBank accession: KU724109]. It encoded a protein with 1,198 amino acids and a calculated molecular mass of 131.9 kDa (Figure 2). A phenogramic analysis confirmed the identity of the deduced PMCA sequence, as it was grouped separately from SPCA and SERCA (Figure 3). The apical PMCA2w/b of *Homo sapiens* and the PMCA of *T. squamosa* comprised more amino acid residues at the splice site A between TM2 and TM3 than the basolateral PMCA2z/b of *H. sapiens* and the PMCA of the hard coral *S. pistillata* (Figure 2). Specifically, at the splice site A, PMCA2w/b of *H. sapiens* had an insert of 45 amino acids (Gly303-Lys347) while the PMCA of *T. squamosa* had an insert of 36 amino acids (Asp285-Asn320), as compared to the PMCA2z/b of *H. sapiens* (Figure 2).

### Subcellular localization of PMCA in the inner mantle

**Figure 4:**
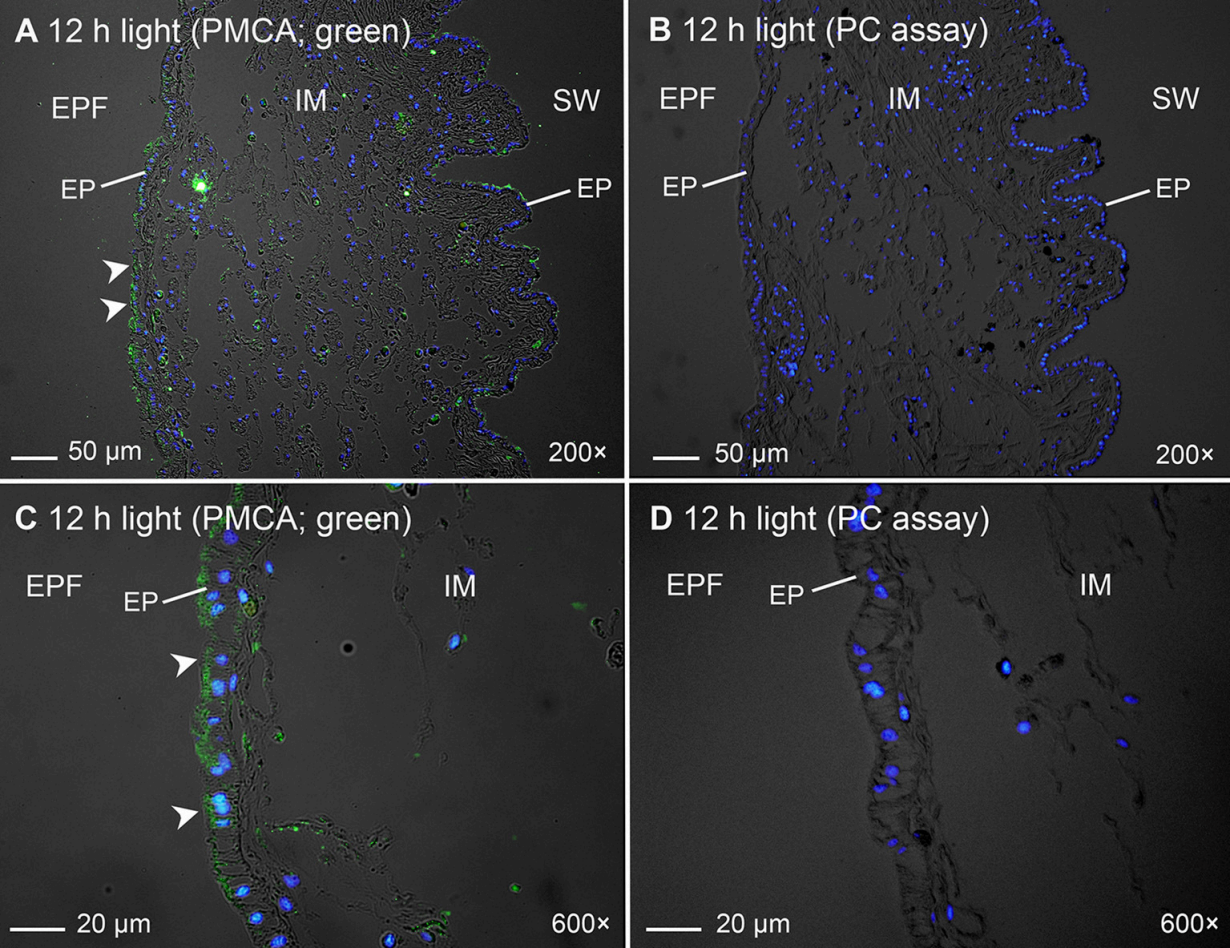
Validation of immunofluorescence of Plasma Membrane Ca^2+^-ATPase (PMCA)-labeling using a custom-made anti-PMCA antibody raised against the PMCA of *Tridacna squamosa* by a peptide competition test (PC). Immunofluorescent localization of PMCA in the inner mantle (IM) of *T. squamosa* exposed to 12 h of light using anti-PMCA antibody **(A,C)**, or anti-PMCA antibody pre-incubated with the immunizing peptide in the PC **(B,D)**. Anti-PMCA immunofluorescence is shown in green with nuclei counterstained with DAPI in blue, and overlaid with differential interference contrast images. Arrowheads in **(A,C)** show apical staining of PMCA on the epithelium (EP) facing the extrapallial fluid (EPF) compared to the lack of PMCA staining in the PC **(B,D)**. SW, seawater. Magnification: 200 × for **(A,B)**; 600 × for **(C,D)**.

**Figure 5:**
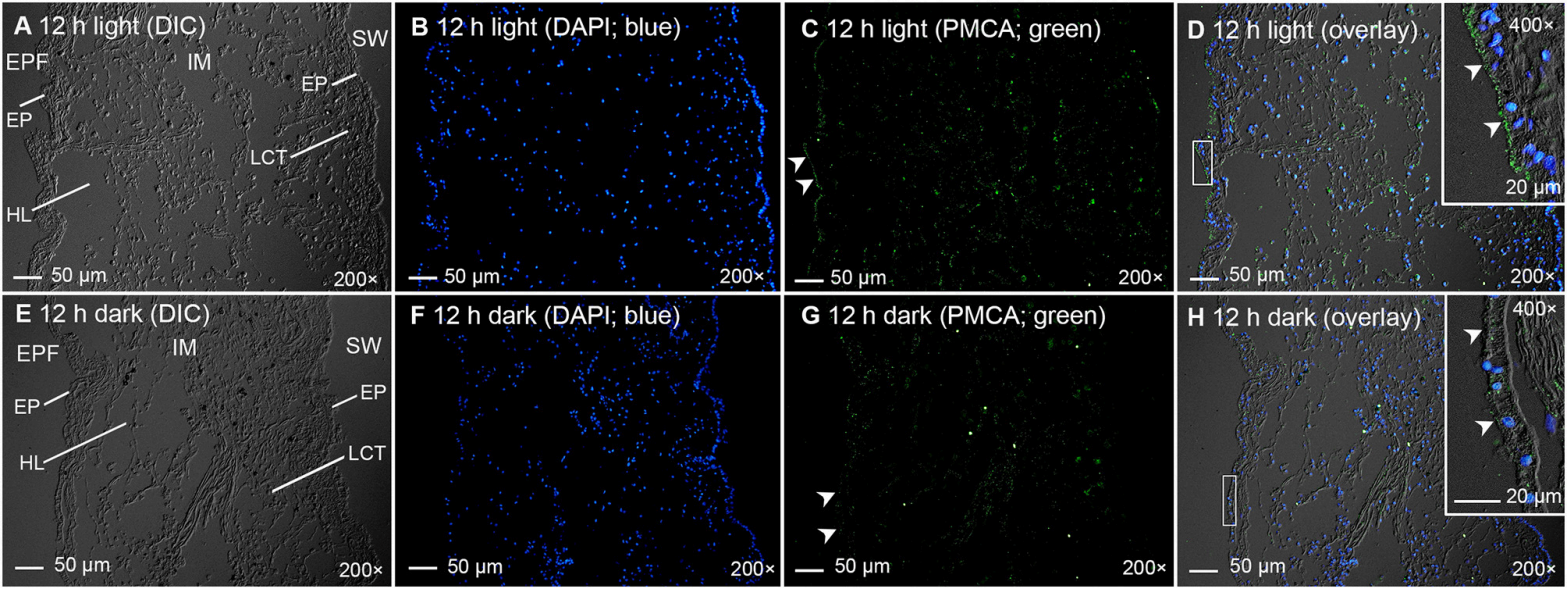
Immunofluorescence microscopy of Plasma Membrane Ca^2+^-ATPase (PMCA) in the inner mantle of *Tridacna squamosa* using a custom-made anti-PMCA antibody raised against its PMCA. Immunofluorescent localization of PMCA in the inner mantle (IM) of *T. squamosa* exposed to 12 h of light **(A–D)** or 12 h of darkness (**E–H**; control). The differential interference contrast images (DIC) labeled with different cellular structures are shown **(A,E)**. The nuclei are counterstained with DAPI in blue **(B,F)**. Anti-PMCA immunofluorescence is shown in green **(C,G)**. All channels (green and blue) are merged and overlaid with DIC **(D,H)**. Arrowheads in **(C)** show more extensive apical staining of PMCA on the epithelium (EP) of the IM facing the extrapallial fluid (EPF) as compared to **(G)**. Arrowheads in the insets of **(D,H)** denote more extensive apical staining of PMCA on the EP of the IM facing the EPF in **(D)** compared to **(H)**. Weak and uneven labeling was observed on the EP of the IM facing the seawater (SW) in **(C,G)**. HL, haemolymph; LCT, loose connective tissues. Magnification: 200 × for **(A–H)**; 400 × for insets of **(D,H)**. Reproducible results were obtained from five clams exposed to light and five clams kept in darkness. Results obtained through quantification of apical immunofluorescence of experimental and control clams are reported in the text.

Immunofluorescence microscopy revealed that the PMCA of *T. squamosa* had an apical localization at the shell-facing epithelium of the inner mantle (Figures 4A,C, 5D,H). The specificity of the anti-PMCA antibody and the validity of the PMCA-immunolabeling were verified by the peptide competition test (Figures 4B,D). While the apical PMCA was localized ubiquitously to the shell-facing epithelium (Figures 5C,D), relatively weak immunofluorescence was detected unevenly along the apical membrane of the seawater-facing epithelium of the inner mantle (Figure 5C). Only faint and scattered immunofluorescence was observed among the loose connective tissues surrounding the haemolymph inside the inner mantle.

### Quantitative immunofluorescence microscopy

Twelve hours of illumination led to an apparent increase in the immunofluorescent-labeling by the custom-made anti-PMCA antibody along the apical membrane of the shell-facing epithelium of the inner mantle (Figures 5A–D) as compared to the control kept in darkness for 12 h (Figures 5E–H). Subsequently, a quantification (integrated density) of immunofluorescence of the apical lining of the shell-facing epithelium of the inner mantle by ImageJ confirmed that the PMCA-staining of the former (1,312 ± 152; *N* = 5) was significantly greater (*P* < 0.05; ~2-fold) than that of the latter (647 ± 103; *N* = 5). A greater integrated density was obtained when the area of quantification was extended to include entire epithelial cells of the shell-facing epithelium, and the integrated density recorded from clams exposed to light for 12 h (2100 ± 230; *N* = 5) was also significantly greater (*P* < 0.05; ~1.7-fold) than that from the control (1,221 ± 189; *N* = 5).

### Effects of light exposure on the *PMCA* transcript level

**Figure 6:**
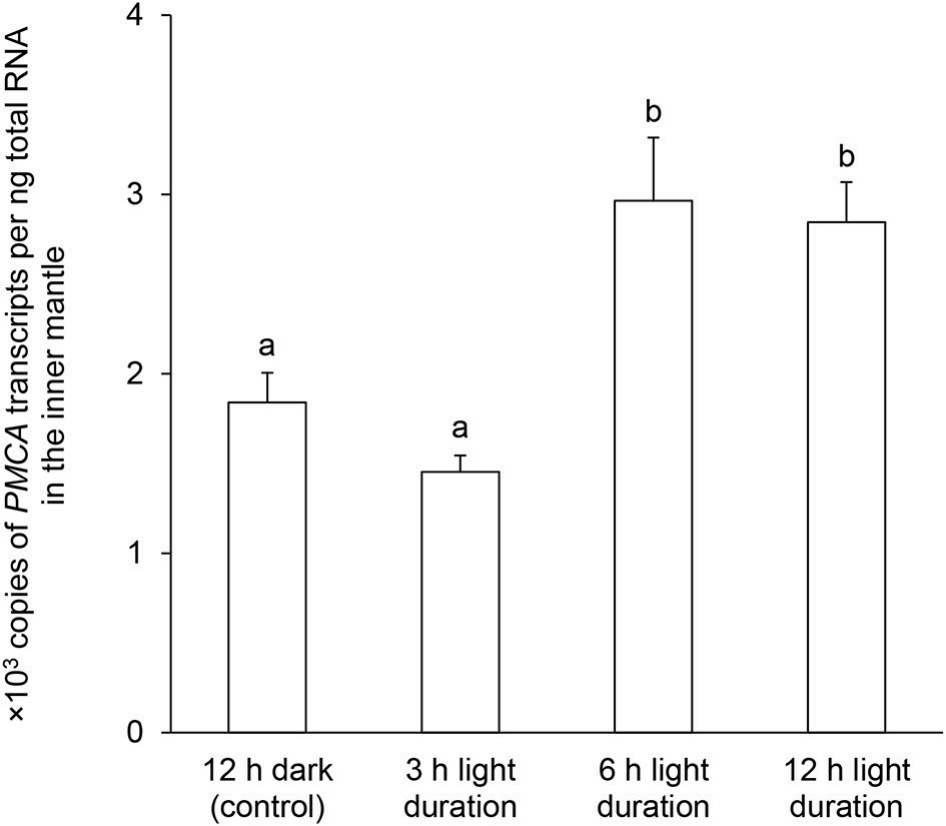
Effects of light on the mRNA expression levels of *Plasma Membrane Ca*^2+^*-ATPase* (*PMCA*) in the inner mantle of *Tridacna squamosa*. Absolute quantification (x 10^3^ copies of transcripts per ng total RNA) of *PMCA* transcripts in the inner mantle of *T. squamosa* exposed to 12 h of darkness (control) or 3, 6, or 12 h of light. Results represent means + S.E.M. (*N* = 5). Means not sharing the same letter are significantly different (*P* < 0.05).

The *PMCA* transcript level in the inner mantle of *T. squamosa* exposed to light for 6 or 12 h was significantly greater (~1.5-fold; *P* < 0.05) than that of the control kept in darkness for 12 h (Figure 6).

### Effects of light exposure on the PMCA protein abundance

**Figure 7:**
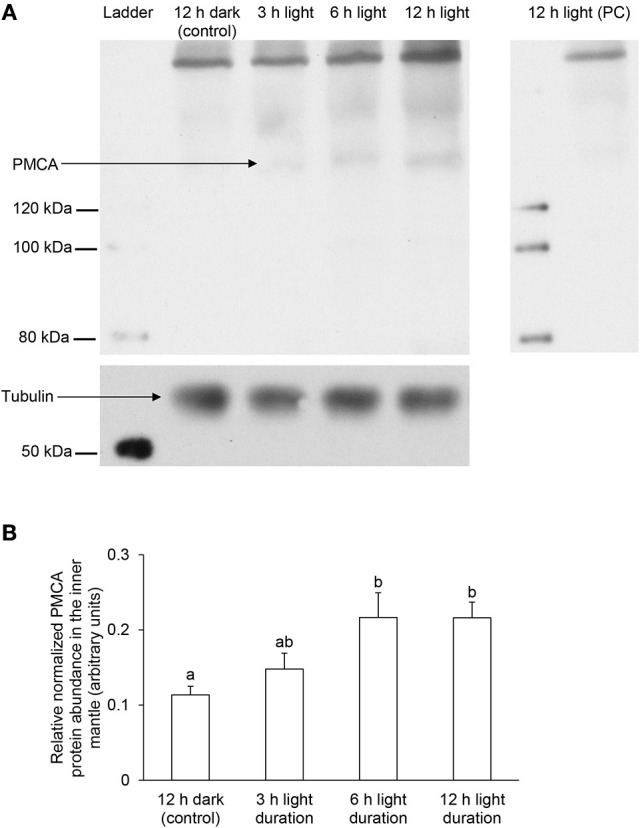
Effects of light on the protein abundances of Plasma Membrane Ca^2+^-ATPase (PMCA) in the inner mantle of *Tridacna squamosa* based on Western blotting using a custom-made anti-PMCA antibody raised against its PMCA. Protein abundances of PMCA in the inner mantle of *T. squamosa* exposed to 12 h of darkness (control) or 3, 6, or 12 h of light. Samples of inner mantle were homogenized in a buffer containing 1% sodium deoxycholate to extract all membrane proteins. **(A)** Examples of immunoblot of PMCA with tubulin as the reference protein, and peptide competition test (PC) to validate the specificity of the anti-PMCA antibody. **(B)** The intensity of the PMCA band for 100 μg protein was normalized with respect to that of tubulin. Results represent means + S.E.M. (*N* = 3). Means not sharing the same letter are significantly different (*P* < 0.05).

Using the custom-made polyclonal anti-PMCA antibody raised against the PMCA of *T. squamosa*, Western blotting revealed a band of interest at ~150 kDa (Figure 7A; see Figure S1 for the whole immunoblot), which was close to the estimated molecular mass of PMCA of *T. squamosa*. Additionally, there was a broad band at ~250 kDa, which could represent dimers as PMCA is known to undergo oligomerization (Levi et al., 2000). A peptide competition test on the sample obtained from clams exposed to light for 12 h eliminated the ~150 and ~250 kDa bands, confirming them as the targeted antigen (Figure 7A). The intensity of the band at ~150 kDa increased significantly (*P* < 0.05) in samples of the inner mantle from clams exposed to light for 6 or 12 h as compared with the control (Figure 7B), which corroborated the quantitative results of immunofluorescence microscopy, and revealed a progressive increase in the protein abundance of PMCA in the inner mantle of *T. squamosa* during the 12-h period of light exposure.

However, results obtained with the custom-made polyclonal anti-PMCA antibody raised against the PMCA of *T. squamosa* also showed a strong band near the origin with unknown identity (Figure 7A). Hence, it became essential to employ the commercially available monoclonal anti-PMCA antibody 5F10, which recognizes an epitope of 60 amino acids (residues 724-783 of the human erythrocyte PMCA), to corroborate the Western blotting and immunofluorescence microscopy results.

### Western blotting and immunofluorescence microscopy using the monoclonal anti-PMCA antibody 5F10

**Figure 8:**
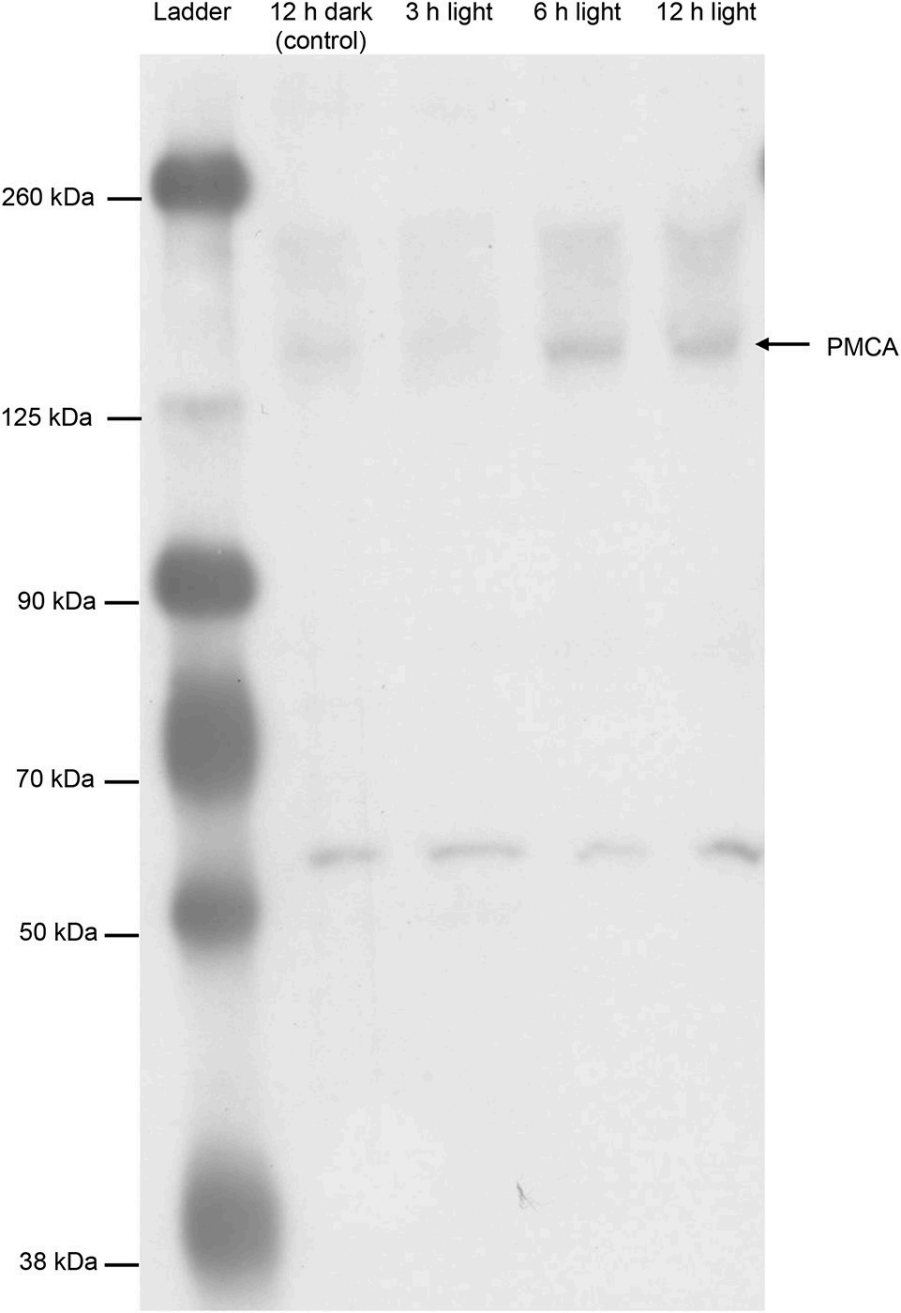
Western blotting of Plasma Membrane Ca^2+^-ATPase (PMCA) from the inner mantle of *Tridacna squamosa* using a commercially available monoclonal anti-PMCA antibody, 5F10. An immunoblot showing the protein abundances of PMCA in the inner mantle of *T. squamosa* exposed to 12 h darkness (control) or 3, 6, or 12 h of light using 5F10 which is known to detect all four known isoforms of PMCA.

The epitope sequence of 5F10 shared ~70% similarity with the related sequence in the PMCA of *T. squamosa*. Western blotting using 5F10 displayed a band of interest at ~150 kDa, although there were also one distinct band at 55 kDa and some other bands of higher molecular masses (Figure 8). As no pre-immunization peptide was available for 5F10, the identity of the ~150 kDa band could not be verified by a peptide competition test. Nevertheless, similar to results obtained with the custom-made polyclonal anti-PMCA antibody raised against the PMCA of *T. squamosa*, the intensity of the ~150 kDa band from the inner mantle of clams exposed to light for 6 or 12 h were substantially greater than that from the control (Figure 8).

**Figure 9:**
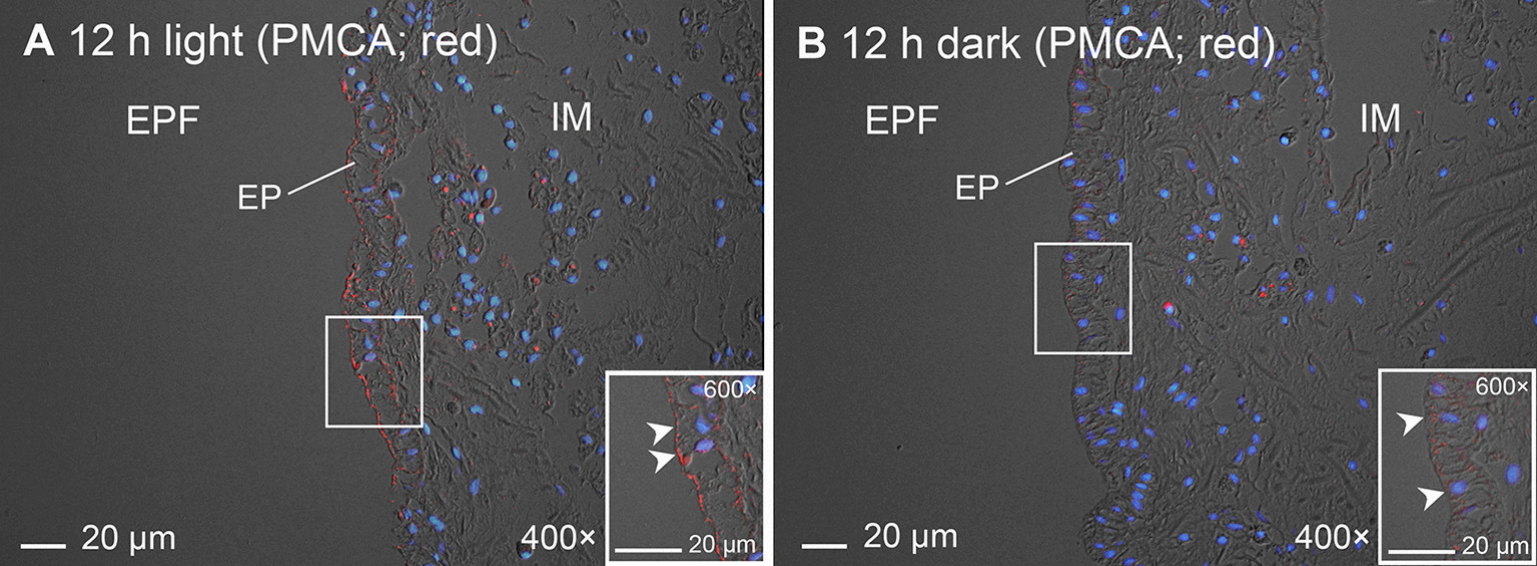
Immunofluorescence microscopy of Plasma Membrane Ca^2+^-ATPase (PMCA) in the inner mantle of *Tridacna squamosa* using a commercially available monoclonal anti-PMCA antibody, 5F10. Immunofluorescence labeling of PMCA in the inner mantle of *T. squamosa* exposed to 12 h of light **(A)** or 12 h of darkness (**B**; control) using 5F10. Anti-PMCA immunofluorescence (red) is merged with DAPI nucleic acid stain (blue) and the differential interference contrast image. Arrowheads in insets show more intense apical anti-PMCA immunofluorescence on the shell-facing epithelium (EP) of the inner mantle (IM) in touch with the extrapallial fluid (EPF) in the IM of *T. squamosa* exposed to light for 12 h as compared with the control kept in darkness. Magnification: 400 × for **(A)** and **(B)**, both with insets at 600 ×.

In addition, immunofluorescence microscopy using 5F10 confirmed both the apical localization of PMCA in the shell-facing epithelium of the inner mantle of *T. squamosa* and the stronger apical immunofluorescent-labeling of the shell-facing epithelium from clams exposed to light for 12 h as compare to the control kept in darkness (Figure 9), corroborating results obtained using the custom-made polyclonal anti-PMCA antibody.

## Discussion

### PMCA of *T. squamosa* comprises an apical targeting element

The PMCA of *T. squamosa* comprised 10 predicted TMs (TM1–TM10), each containing 16 to 26 amino acids. It also consisted of four main cytosolic domains, which included the aspartic acid-lysine-threonine-glycine-threonine-lysine-threonine (DKTGTLT) motif, the ATP/FITC-binding domain, the signature calmodulin-binding domain, and the threonine-glycine-glutamic acid-serine (TGES) motif (Di Leva et al., 2008). Like all P-type ATPases, the intracellular loop between TM4 and TM5 of the PMCA of *T. squamosa* contained the catalytic core of the pump which has a phosphorylated aspartate residue (Asp508, based on the alignment in Figure 1) and a conserved lysine residue (Lys634) involved in the binding with ATP. As PMCA and SERCA are members of the P2-ATPase family, they have similarities in Ca^2+^ affinity, organization of catalytic domains, and membrane topography (Carafoli and Brini, 2000). A comparison between PMCA of *T. squamosa* and SERCA1a of *Oryctolagus cuniculus* revealed five homologous amino acid residues (Val461, Glu466, Asn909, Asn937, and Asp941) which could be involved in Ca^2+^ binding (Toyoshima et al., 2000).

The physiological functions of PMCA isoforms are defined by their localization to specific membrane compartments, and the subcellular localizations of a specific PMCA isoform is dependent on the amino acid residues in the splice site A as well as some other factors (e.g., interacting proteins). In PMCA, alternative splicing can occur at splice site A, which is located in the first intracellular loop between TM2 and TM3, and splice site C, which is located at the COOH terminal tail. In mammalian PMCA2, splicing at splice site A with the insertion of three discrete exons can generate four types of splice variants: PMCA2w (with the insertion of 45 amino acids), PMCA2x (with the insertion of 14 amino acids), PMCA2y (with the insertion of 31 amino acids), and PMCA2z (no insertion). The amino acid residues in splice site A imposes dominant membrane targeting information for PMCA2 of *H. sapiens*. PMCA2x/b and PMCA2z/b, which contain only 14 and 0 spliced-in residues (Chicka and Strehler, 2003; Padányi et al., 2010), respectively, are mostly located at the basolateral membrane of polarized epithelia (Enyedi and Strehler, 2007). By contrast, PMCA2w/b, which contains a w-splice insert of 45 amino acids (residues 303-347; Figure 2), has an apical localization in epithelial cells. By transplanting the PMCA2 w-insert into the equivalent position of PMCA4b and then determining the localization of the chimeric PMCA4(2w)/b in polarized Madin-Darby canine kidney cells, the w-insert of PMCA2 is confirmed to act as an autonomous apical targeting element (Antalffy et al., 2012). Apparently, the PMCA2 w-insert can guide the transporter to the apical membrane provided that it contains >30 amino acids (Enyedi and Strehler, 2007). Even a scrambled amino acid sequence of >30 residues, which differ completely from the genuine PMCA2 w-insert, is effective in directing the transporter to the apical membrane (Enyedi and Strehler, 2007). Notably, in rat, a PMCA2y splice variant containing 31 spliced-in residues at the splice site A (Adamo and Penniston, 1992) also displays an apical localization in the hair cells of the inner ear (Grati et al., 2006). Somewhat similar to the apical PMCA2y of rat and the apical PMCA2w/b of humans, the PMCA of *T. squamosa* contained an additional 36 amino acids at the splice site A between TM2 and TM3 as compared to the basolateral PMCA2z/b. Therefore, based on the molecular characteristics of PMCA2y and PMCA2w/b, it can be deduced that PMCA of *T. squamosa* should have an apical localization in the mantle epithelium.

### PMCA is localized to the apical membrane of the shell-facing epithelium of the inner mantle of *T. squamosa*

Indeed, immunofluorescence microscopy confirmed that PMCA was localized ubiquitously along the apical membrane of the shell-facing epithelium of the inner mantle of *T. squamosa*. Of note, the inner mantle is in direct contact with the extrapallial fluid where light-enhanced calcification occurs (Sano et al., 2012). As immunofluorescent PMCA-labeling was weak and uneven along the seawater-facing epithelium, and also faint and scattered among the loose connective tissues of the inner mantle, it is logical to deduce that the apical PMCA of the shell-facing epithelium plays an important role in shell formation in *T. squamosa*. With its apical localization, PMCA is poised to supply Ca^2+^ from the inner mantle epithelial cells to the extrapallial fluid, although the possible involvement of other types of Ca^2+^ transporter (e.g., Na^+^/Ca^2+^ exchanger) cannot be ruled out. Nonetheless, PMCA functions as an obligatory Ca^2+^/H^+^ exchanger with a probable stoichiometry of 1:1 (Salvador et al., 1998). Hence, its apical localization at the shell-facing epithelium of the inner mantle would facilitate not only the export of Ca^2+^, but also the removal of some of the H^+^ generated in the extrapallial fluid during CaCO_3_ precipitation. This would complement the increased removal of H^+^ as ${\text{NH}}_{4}^{+}$ during light exposure, underscoring the importance of raising the pH of the extrapallial fluid during light-enhanced calcification (Ip et al., 2006, 2015).

Scleractinian corals also harbor symbiotic zooxanthellae and undergo light-enhanced calcification. The transepithelial movement of Ca^2+^ through the calicoblastic epithelium to the site of skeleton formation may involve a Ca^2+^-ATPase (Allemand et al., 2011), as Ca^2+^-ATPase activity has been detected in membrane-enriched fractions from the coral *Galaxea fascicularis* (Ip et al., 1991). PMCA has been postulated to have an apical localization in calicoblastic cells of scleractinian corals, transporting Ca^2+^ directly to the subcalicoblastic fluid where calcification occurs (Zoccola et al., 2004; Davy et al., 2012). While a *PMCA* has been cloned and sequenced from *S. pistillata*, it lacks a functional w-insert equivalent (Figure 2) and is allegedly expressed in the aboral tissue (Zoccola et al., 2004). Furthermore, contrary to the early postulation, this PMCA has been localized to the cytoplasm of calicoblastic cells of *S. pistillata* by immunofluorescence microscopy (Barott et al., 2015). Similarly, PMCA has been reportedly localized to the cytoplasm of the cells in the shell-facing mantle epithelium of the non-symbiotic bivalve, *Anodonta cygnea*, which is incapable of light-enhanced calcification (Lopes-Lima et al., 2008). Of note, PMCAs are by definition not localized in the cytoplasm; therefore, those information in the literature should be interpreted with caution, and the possibility of an apical Ca^2+^-ATPase being involved in calcification in scleractinian corals and non-symbiotic bivalves cannot be excluded completely. Nonetheless, the discovery of the apical localization of PMCA in the shell-facing epithelium of the inner mantle adjacent to the extrapallial fluid of *T. squamosa* is novel, and there could indeed be differences in mechanisms of light-enhanced calcification between scleractinian corals and giant clams, and in mechanisms of shell formation (calcification) between giant clams and other non-symbiotic bivalves.

### The transcript level and protein abundance of *PMCA*/PMCA in the inner mantle of *T. squamosa* are light-dependent

As *T. squamosa* engages in light-enhanced calcification (Sano et al., 2012), it was obviously important to examine whether the expression of *PMCA*/PMCA in the inner mantle are light-inducible. Indeed, 12 h of illumination resulted in a significantly stronger immunolabeling along the apical membrane as well as in the whole cells of the shell-facing epithelium of the inner mantle. In addition, there were significant increases in the transcript level and protein abundance of *PMCA*/PMCA in the inner mantle of *T. squamosa* exposed to light as compared with the control. These results indicate that the transcription and the translation of *PMCA*/PMCA in the inner mantle of *T. squamosa* can be up-regulated by light, denoting PMCA as one of the rate-limiting transporters involved in light-enhanced calcification.

Sano et al. (2012) reported that the shell of *T. derasa* displayed diurnal variations of Sr/Ca ratio, and proposed Ca^2+^-ATPase as the dominant mechanism that caused Sr/Ca variability. Therefore, the apical localization of PMCA along the shell-facing epithelium of the inner mantle of *T. squamosa* and the upregulation of its gene and protein expression by light offer not only a novel explanation of light-enhanced calcification in giant clams, but also a mechanistic explanation of the Sr/Ca variability in their shells. Noticeably, Ip et al. (2015) were unable to detect any light-enhancing effect on the Ca^2+^-ATPase activity from the inner mantle *T. squamosa*. As the enzyme activity reported by Ip et al. (2015) was attributable to all the P2-ATPases, including PMCA, SERCAs, and SPCAs, it would not be possible to detect an increase in the comprehensive Ca^2+^-ATPase activity if PMCA was only a minor component of all P2-ATPases in the inner mantle, or if the other P2-ATPases in the inner mantle were not light-dependent, or both.

### General implications to light-enhanced calcification in alga-invertebrate association

Several hypotheses have been proposed to explain light-enhanced calcification in scleractinian corals (see Tambutté et al., 2011 for a review), which are presumably also pertinent to giant clams. Firstly, as calcification produces H^+^ which may react with ${\text{HCO}}_{3}^{-}$ to form CO_2_, photosynthesis in the symbiotic zooxanthellae can drain CO_2_ from the calcification site and thus favors CaCO_3_ precipitation. Secondly, the removal of substances which inhibit calcification, such as phosphates, by the photosynthesizing symbiotic zooxanthellae may enhance calcification. Thirdly, in light, zooxanthellae release photosynthate to the host and fuels the generation of ATP, which may support the increased rate of calcification. Fourthly, during photosynthesis, the release of O_2_ by zooxanthellae may promote the host's aerobic energy metabolism and augment calcification. Fifthly, zooxanthellae undergoing photosynthesis may supply the host with precursors needed for the synthesis of organic matrix required for shell formation. As the operation of PMCA is energetically supported by ATP hydrolysis, our results denote that light-enhanced calcification in *T. squamosa* is attributable indirectly to the release of photosynthate and O_2_ from zooxanthellae, which augments aerobic energy metabolism in the host to support the light-enhanced PMCA activity.

The discovery of light having an enhancing effect on the gene and protein expression levels of certain transporters/enzymes (PMCA, this study; NHE3-like transporter, Hiong et al., 2017a; GS, Hiong et al., 2017b) in the inner mantle and ctenidium of *T. squamosa* signifies the possible effects of light on the host's mechanisms engaging directly or indirectly in the calcification process of alga-invertebrate associations including scleractinian corals. Actually, several studies have already suggested that calcification in corals can also be enhanced directly by light. Using microsensors, Al-Horani et al. (2003) demonstrated that exposure to light can induce increases in pH and [Ca^2+^] in the calcifying fluid of corals. Insolation also stimulates the uptake of dissolved inorganic carbon and Ca^2+^ by the calicoblastic cells adjacent to the extracellular calcifying fluid (Mueller, 1984; Furla et al., 2000). Recently, Cohen et al. (2016) have reported the enhancement of calcification in the corals, *Porites lutea* and *Acropora variabilis*, by blue light which has low efficiency in inducing algal photosynthesis. They have suggested that blue light photoreceptors in coral tissue can be the light sensor which activates a Ca^2+^/H^+^ antiporter type of Ca^2+^-ATPase involved in blue light-enhanced calcification (Cohen et al., 2016). Indeed, Marshall (1996) reported that light-enhanced calcification in *G. fascicularis* was inhibited by the Ca^2+^-ATPase inhibitor Ruthenium red, albeit the concentration of inhibitor used was too high to achieve PMCA-specificity. Taken altogether, it can be concluded that the possibility of scleractinian corals having some sort of light-dependable Ca^2+^ transporter cannot be ignored.

### The metabolic advantages of living in symbiosis with zooxanthellae

Daily up- and down-regulations of the gene and protein expression of *PMCA*/PMCA in *T. squamosa* are seemingly energy-wasteful, as transcription and translation processes involve energy expenditure. However, unlike free-living animals, giant clams live in symbiosis with zooxanthellae, and an estimated 90–95% of the carbon fixed daily by the zooxanthellae during photosynthesis is translocated to the host (Muscatine et al., 1983; Edmunds and Davies, 1986; Davies, 1991). The compounds translocated include glycerol, glucose, and amino acids (Muscatine, 1967; Streamer et al., 1988), and the quantity of translocated carbon is sufficient to meet the daily energy and growth requirements of the host clam (Fisher et al., 1985; Klumpp et al., 1992; Klumpp and Griffith, 1994; Hawkins and Klumpp, 1995). Conceivably because of that, *T. squamosa* could afford energetically to regulate light-inducible processes through transcriptional and translational changes. A corollary of the apparent light-dependent diurnal transcriptional and translational regulation of PMCA (this study), GS (Hiong et al., 2017b), and NHE3-like transporter (Hiong et al., 2017a) is that the turnover of the relevant transcripts and proteins must be rather rapid. Therefore, efforts should be made in the future to determine the half-life of these proteins and to examine the regulatory mechanisms of their turnover.

### Can symbiotic zooxanthellae act as a light-sensor to the clam host?

The inner mantle of *T. squamosa* is whitish in color and lacks pigment of the host origin. Non-pigmented animal tissues are usually not light-responsive, because pigments that can be oxidized by light are typically essential components of light-specific sensors (Helten et al., 2007). Hence, it is intriguing that the transcription and translation of *PMCA*/PMCA in the inner mantle of *T. squamosa* can be light-dependent. As giant clams possess siphonal eyes (Wilkens, 1986), it is possible that light is detected directly by the host clam, with neuronal or hormonal signals being subsequently transmitted to other parts of the body. It is also possible that the symbiotic zooxanthellae act as “light-sensing” elements for the clam host (Ip et al., 2015; Hiong et al., 2017a,b). Zooxanthellae are known to possess both opsin, which is a photoreceptor protein, and eye-spots comprising crystalline clusters of uric acid (Yamashita et al., 2009). Although the inner mantle harbors only a small quantity of zooxanthellae, which are located mainly in a slightly brownish area near the hinge shaded from light, plenty of zooxanthellae reside in the outer mantle which can be extended beyond the edge of the shell-valve during insolation. As proposed previously (Ip et al., 2015; Hiong et al., 2017a,b), in response to light, zooxanthellae residing in the outer mantle may produce some sort of signaling molecules and release them to the extracellular fluid of the clam host. Upon reaching the inner mantle, these signaling molecules may activate the transcription and/or translation of certain genes/proteins (e.g., *PMCA*/PMCA) essential for light-enhanced calcification in the epithelial cells. If proved to be correct, this proposition would imply that the symbiotic cooperation between the symbiotic zooxanthellae and the host clam entails not only the transfer of inorganic and organic substances but also the transmission of signals and information between them.

## Accession codes

The *PMCA* nucleotide sequence from inner mantle of *T. squamosa* has been deposited in GenBank [Accession: KU724109].

## Author contributions

YI designed the study and wrote the manuscript. KH, EG, MB, CC, and BC performed the experiment. KH, EG, MB, CC, BC, SC, and YI analyzed the data. WW and SC contributed reagents, materials and analysis tools. All authors reviewed the manuscript.

### Conflict of interest statement

The authors declare that the research was conducted in the absence of any commercial or financial relationships that could be construed as a potential conflict of interest.
