# Supplementary material for: The Whitish Inner Mantle of the Giant Clam, Tridacna squamosa, Expresses an Apical Plasma Membrane Ca2+-ATPase (PMCA) Which Displays Light-Dependent Gene and Protein Expressions
Source: Front Physiol. 2017 Oct 10;8:781. doi: 10.3389/fphys.2017.00781 (PMC5641333; doi:10.3389/fphys.2017.00781)
Supplement: Supplementary file 1 [file Image1.PDF]

Figure S1. Effects of light on the protein abundances of Plasma Membrane Ca^{2+}-ATPase (PMCA) in the inner mantle of *Tridacna squamosa*. Protein abundances of PMCA in the inner mantle of *T. squamosa* exposed to 12 h of darkness (control) or 3, 6 or 12 h of light. Samples of inner mantle were homogenized in a buffer containing 1% sodium deoxycholate to extract all membrane proteins. Examples of immunoblot of PMCA, PMCA with immunizing peptide, and tubulin as the reference protein.

Figure S1

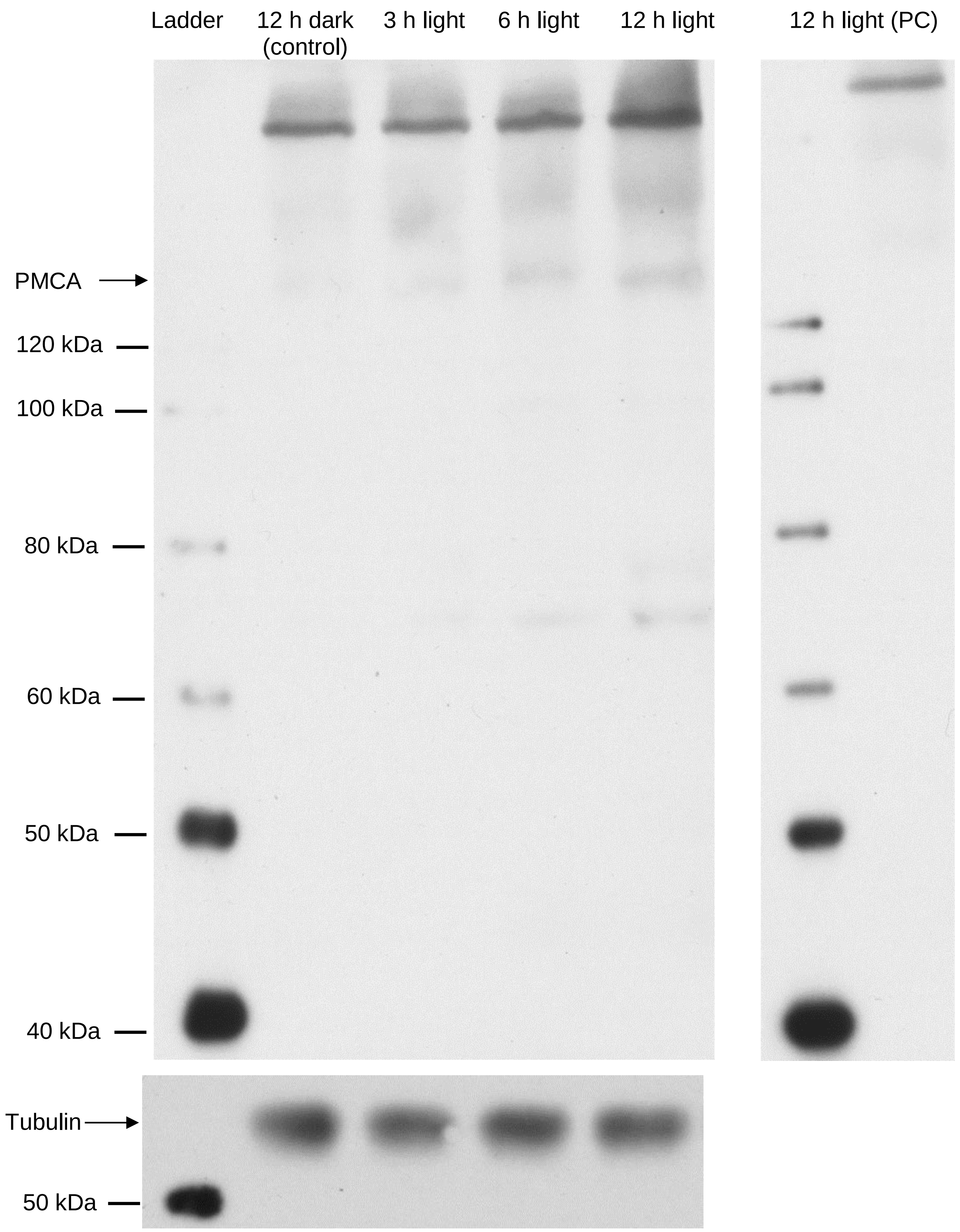
Ladder
12 h dark (control)
3 h light
6 h light
12 h light
12 h light (PC)
PMCA
120 kDa
100 kDa
80 kDa
60 kDa
50 kDa
40 kDa
Tubulin
50 kDa
